# Individual-based modelling and control of bovine brucellosis

**DOI:** 10.1098/rsos.180200

**Published:** 2018-05-02

**Authors:** Erivelton G. Nepomuceno, Alípio M. Barbosa, Marcos X. Silva, Matjaž Perc

**Affiliations:** 1 Control and Modelling Group (GCOM), Department of Electrical Engineering, Federal University of São João del-Rei, São João del Rei, Brazil; 2 Department of Electrical Engineering, Centro Universitário Newton Paiva, Belo Horizonte, Brazil; 3 Epidemiological Laboratory, Department of Preventive Veterinary Medicine, Federal University of Minas Gerais, Belo Horizonte, Brazil; 4 Faculty of Natural Sciences and Mathematics, University of Maribor, Koroška cesta 160, 2000 Maribor, Slovenia; 5 CAMTP—Center for Applied Mathematics and Theoretical Physics, University of Maribor, Mladinska 3, 2000 Maribor, Slovenia; 6 Complexity Science Hub, Josefstädterstraße 39, 1080 Vienna, Austria

**Keywords:** bovine brucellosis, individual-based model, mathematical epidemiology

## Abstract

We present a theoretical approach to control bovine brucellosis. We have used individual-based modelling, which is a network-type alternative to compartmental models. Our model thus considers heterogeneous populations, and spatial aspects such as migration among herds and control actions described as pulse interventions are also easily implemented. We show that individual-based modelling reproduces the mean field behaviour of an equivalent compartmental model. Details of this process, as well as flowcharts, are provided to facilitate the reproduction of the presented results. We further investigate three numerical examples using real parameters of herds in the São Paulo state of Brazil, in scenarios which explore eradication, continuous and pulsed vaccination and meta-population effects. The obtained results are in good agreement with the expected behaviour of this disease, which ultimately showcases the effectiveness of our theory.

## 1. Introduction

Several researchers seek to develop mathematical models that can contribute to understanding the spread and prevalence of bovine brucellosis [1–6]. The mathematical modelling is commonly employed in epidemiology using ordinary differential equations (ODE) and the compartmental approach, such as the compartmental model SIR (susceptible–infected–recovered), proposed by Kermack & McKendrick [7]. In such model, important characteristics can be analysed, among them: the mass action principle and the existence of a threshold for eradication of the disease.

Even though compartmental models have been widely studied in the last decades, some important recent contributions have been published [8–10]. One of these applications has been in the mathematical modelling of bovine brucellosis. Dias [11] has presented an adaptation of [12] for the mathematical modelling of the propagation of bovine brucellosis (also seen in [13]). In this approach, these authors use compartmental models. There are other interesting works, in which many authors develop logistic regression models to analyse prevalence and risk studies of brucellosis [1,6,14]. More recently, the authors in [3] have employed a compartmental model to measure the impact of the combined use of these two vaccines in reducing the prevalence of bovine brucellosis. This combined use has been shown to be a good alternative to a significant decrease in the prevalence of bovine brucellosis in a shorter period.

Although many papers describe expressive developments on the modelling of bovine brucellosis, there has been less attention to develop individual-based model (IBM) to describe such disease. This approach is an obvious superior alternative when compared to compartmental models, as the system can be seen from a network perspective. There is great attention to investigate the spread of infectious diseases in networks, where spatial and heterogenous population can be considered [15–19]. This perspective is highly important; as has been already pointed out in [20], uniform vaccination is very inefficient for disease eradication in heterogeneous networks. The need of such IBMs for epidemiology systems is also cleared established in [21–24]. Regarding the investigation on bovine brucellosis, there are reasons that compartmental models are not totally efficient to understand the dynamics of bovine brucellosis. For instance, it has been noticed by Veloso *et al.* [14] that larger herds present higher risks of prevalence of bovine brucellosis. The same remark has been found in [24], where a generic IBM has been investigated. Besides that, compartmental models cannot evaluate spatial issues of disease transmission, also lost in mobility of animals, which affects their rate of disease transmission to other animals that are distant from each other.

One of the first attempts to describe the dynamics of bovine brucellosis in cattle herds using IBM can be seen in [25]. There are a few other works that use IBM to model brucellosis as seen in [26,27]. However, these works have not considered important aspects that influence the spread of bovine brucellosis, such as the change in the number of individuals in herds, and traffic of animals among herds. Silva [25] has used only the vaccination as action of control, leaving aside the isolation and slaughter, which also are important actions of control. Moreover, as the compartmental models are more intuitive, it would be benefit a consistent explanation of the intrinsic relationship between these two strategies, which are not clearly stated in works such as [26,27]. This connection with the phenomenon under investigation is an important aspect, as it is not uncommon to have a model that cannot exactly replicate an actual disease, although the results, if well established, may aid decision-makers to design the most effective surveillance or control strategy [27]. In order to correct this weakness, at least partially, this paper presents a stochastic model for bovine brucellosis. It is presented the methodology to develop the model in detail with a strong connection with its counterpart compartmental model. We have investigated three numerical examples using real parameters of herds in São Paulo state (Brazil), which can be summarized in the following three scenarios: (i) eradication; (ii) meta-population effects and (iii) control action. The results show that the IBM may help one to undertake a set of actions to eradicate a disease in farms, such as isolation of infected animals or reduction of the size of population.

## 2. Compartmental model

**Figure 1.**
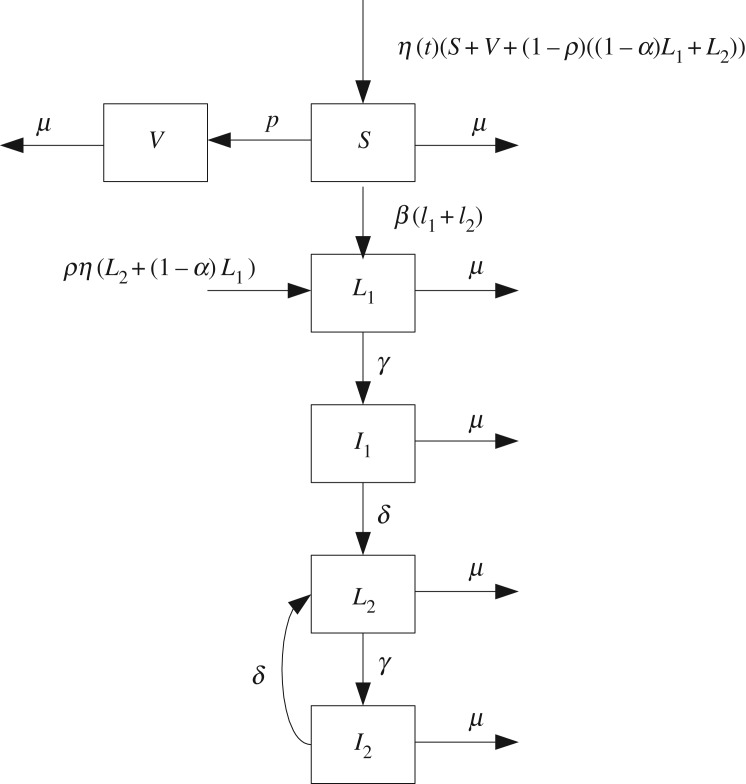
Dynamics of brucellosis in cattle populations described in block diagram. The authors in [13] have used six compartments to describe the dynamics of bovine brucellosis in the population. These compartments are: females susceptible (*S*), vaccinated females (*V*), latent carriers primiparous (*L*_1_), infectious primiparous females (*I*_1_), latent carriers multiparous (*L*_2_) and female infectious multiparous (*I*_2_).

The compartmental model developed by Amaku *et al.* [13] has been proposed to simulate the dynamics of brucellosis in the rearing system for production of milk, if the flock was composed entirely of females. In order to reach the main features of the epidemiological system, the population has been divided into six compartments: females susceptible (*S*), vaccinated females (*V*), latent carriers primiparous (*L*_1_), infectious primiparous females (*I*_1_), latent carriers multiparous (*L*_2_) and female infectious multiparous (*I*_2_), as shown in figure 1.

It was determined by the parameters of the model [13] through data collection and statistical studies according to the epidemiological situation found in the state of São Paulo.

2.1 $$\begin{array}{rl} \frac{dS(t)}{dt} & =[1-p(t)]\eta (t)\{S+V+(1-\rho )[(1-\alpha )L_{1}+L_{2}]\}\\ & \;-\mu S(t)-\beta [I_{1}(t)+I_{2}(t)]S(t),\\ \frac{dL_{1}(t)}{dt} & =\beta [I_{1}(t)+I_{2}(t)]S(t)+\rho \eta (t)[L_{2}(t)+(1-\alpha )L_{1}(t)]-(\gamma +\mu )L_{1},\\ \frac{dI_{1}(t)}{dt} & =\gamma L_{1}-(\delta +\mu )I_{1},\\ \frac{dL_{2}(t)}{dt} & =\delta (I_{1}+I_{2})-(\gamma +\mu )L_{2},\\ \frac{dI_{2}(t)}{dt} & =\gamma L_{2}-(\mu +\delta )I_{2}\\ \;\text{and}\;\frac{dV(t)}{dt} & =p(t)\eta (t)\{S+V+(1-\rho )[(1-\alpha )L_{1}+L_{2}]\}-\mu V. \end{array}\}$$

## 3. Model development

The IBM are simulations based on the global consequences of local interactions of members of the population. For this, we assumed a herd composed of females. Females are more sensitive and remain chronically infected, with a central role in the spread of the disease [22,24,28].

Nepomuceno *et al.* [24] expressed IBM, in which an individual is represented by

3.1 $$I_{m,t}=[C_{1}\;C_{2}\;\cdots \;C_{n}],$$

where 1≤*m*≤*N* is an individual in the population of size *N*, *t* is the moment when an individual has a specific set of features and *C*_*n*_ is a characteristic of the individual. The first feature is its state from the epidemiological point of view. For the first characteristic of bovine brucellosis may be susceptible, vaccinated, a latent, infected 1, 2 or latent infection 2. Other features are the age, duration of the infection, the lag time, sex or any other characteristics that are considered relevant. In turn, a population is represented by

3.2 $$P_{t}={\left[I_{1,t}\;I_{2,t}\;I_{3,t}\;\cdots \;I_{N,t}\right]}^{T},$$

where *I*_*m*,*t*_ is an individual at time *t* and *P* is a matrix *N*×*n*.

### 3.1. Premises and model characteristics

In this section, we describe in more detail, a set of premises used to describe the dynamics of brucellosis in the IBM framework. These premises are as follows.

(1) *Spatial distribution.* The individuals are distributed over a rectangular area. There are two situations: (i) The number of individuals is the same as the number of cells of the rectangular area. In this situation every movement of an individual involves the exchange of position with another individual. (ii) The number of individuals is smaller than the number of cells, so an individual can move to other cells without the need to exchange with other individuals. The second option is more realistic and it is adopted in this work. This is a clear advantage of IBM over the compartmental models based on ODE.

(2) *Population constant*. We have chosen to use a constant population of size *N* for all locations. These considerations have been made to compare with the mean field result of SIR model, but it can be easily relaxed, as has been done in [24].

(3) *Categories of the population*. There are six categories for the population: 0 (susceptible), 1 (vaccinated), 2 (latent primiparous), 3 (infectious primiparous), 4 (latent multiparous) and 5 (infectious multiparous).

(4) *Characterization of the individual*. An individual is characterized by a set of *n* characteristics, where *C*_1_ is the category of individual, *C*_2_ and *C*_3_ are, respectively, the current age and maximum age of the individual in years. *C*_4_ the time the individual is in a latent state primiparous and *C*_5_ the maximum time an individual is in this state. *C*_6_ time in years that the individual is infected primiparous in the state and *C*_7_ the maximum time an individual is in this state. *C*_8_, *C*_9_, *C*_10_ and *C*_11_ are the time that the individual is infected with latent and multiparous, the maximum time an individual becomes infected in a latent state and multiparous females, respectively.

(5) *Change of category*. Once in a category, the individual may move to another category in each instant of time. In this work, we adopted a discrete transition. Transitions can occur in the following ways:

(a) 0, 1, 2, 3, 4, 5 → 0. This means that after a death of an individual, other one is born to maintain a constant population premise, as suggested in 2. If the individual does not die, there may be other transitions, described below.
(b) 0 → 1. A susceptible individual is vaccinated, and this category is changed into 1.
(c) 0 → 2. A susceptible individual may become latent primiparous and move to the category 2.
(d) 2 → 3. A female primiparous, as time passes, can move to the category of infectious primiparous.
(e) 3 → 4. Infectious primiparous can move to category 4, in the course of time.
(f) 4 → 5. A female latent multiparous may become infected multiparous in a time interval.
(g) 3, 5 → 2, 4. Infectious individuals can return to the latent primiparous and multiparous, respectively.

(6) *Statistical distribution*. It has been adopted the exponential distribution, expressed as *m*(*x*)=*μ* *e*^−*μx*^ for mortality and birth. This distribution was also used for the transition from latent infection and given by *l*(*x*)=*γ* *e*^−*γx*^ and *i*(*x*)=*δ* *e*^−*δx*^, respectively. *x* is a random number.

(7) *Infection process*. Each contact between a susceptible and seropositive (or latent infection) individual can cause a new latent individual following a uniform distribution. It is a stochastic process in which *β*% of the individuals have probability to become latent. In IBM, we can change the value of *β* according to individual location or any other feature *C*_*n*_.

(8) *Abortion process*. The process of abortion in female occurs in infected primiparous. However, it has been considered a rate of abortion equal to zero to reduce the complexity of the model. Note that, for a herd where there are no abortions (*α*→0), we have that *η*(*t*)=*μ*, i.e. birth rates and mortality are compensating by keeping a constant population, which is consistent with the premise 2.

(9) *Vaccination process*. Continuous and pulsed vaccination are investigated in the female susceptible individuals *S*. Animals of all ages can be vaccinated without distinction. It was considered that the vaccine protects 100% of vaccinated animals (eligible for vaccination to a certain extent).

### 3.2. Formulation

The formulation of the IBM allows adding various characteristics of individuals, which can make the model more realistic. For bovine brucellosis, according to the assumptions discussed so far, the characteristics of individuals are:

(a) *C*_1_∈[0,1,2,3,4,5]. That is, the individual may be in the state susceptible, vaccinated, latent primiparous, infected primiparous, multiparous and latent infected multiparous, respectively.
(b) *C*_2_ is the individual’s age in years. The age is added *Δt* in each transition.
(c) *C*_3_ is the maximum age at which the individual will live. A birth of an individual is given by:
  3.3 $$C_{3}=-\mu \;\ln(a_{u}),$$
  where *μ* is the life expectancy of the population, around 8 years, and *a*_*u*_ is a random variable with uniform distribution, contained between the values 0 and 1.
(d) *C*_4_ is the time in years that the individual is in a latent state primiparous.
(e) *C*_5_ is the maximum time that the individual is in a latent state primiparous. Similar to the characteristic *C*_3_, the maximum time in which the individual is in a state of latency is obtained by:
  3.4 $$C_{5}=-\gamma \;\ln(a_{u}),$$
  where *γ* is the rate of patients with latent infection.
(f) *C*_6_ is the time in years that the individual is in an infected state primiparous.
(g) *C*_7_ is the maximum time in years, the infected individual is in the state primiparous given by
  3.5 $$C_{7}=-\delta \;\ln(a_{u}),$$
  where *δ* is the rate that determines the period of infection.
(h) *C*_8_, *C*_9_, *C*_10_ and *C*_11_ are characteristics similar to *C*_6_ and *C*_7_, related to the maximum time in which individuals are going to stay in the other features.

*C*_4_ to *C*_11_ are unnecessary for susceptible individuals and vaccinated like *C*_10_ and *C*_11_ are unnecessary for infected individuals latent and multiparous. These cases are considered equal to zero ∀*t*. Tables 1 and 2 illustrate possible transitions arising from the interaction between the animals. The model depicts the change of state of five individuals. The state transitions occur in an interval (*Δt*) of 0.1 years. Some transitions are discussed in detail as follows:

*I*_1,0_→*I*_1,1_. This vaccinated individual dies. In the model is replaced by an individual susceptible to *C*_2_=0 and *C*_3_=7.5.
*I*_2,0_→*I*_2,1_. Became latent primiparous. Its latency period is given by *C*_5_ equal to 0.9 years.
*I*_3,0_→*I*_3,1_. There were no changes in the epidemiological state of that individual.
*I*_4,0_→*I*_4,1_. The latency state that individual ended up. It passed primiparous infected with time of infection (*C*_7_) of 0.7 years.
*I*_5,0_→*I*_5,1_. The individual before infection, returned to the latent multiparous. Its onset is given by *C*_9_ equal to 0.8 years.

**Table 1.** Transitions in an IBM for bovine brucellosis at time *t*=*t*_0_.

*C*_1_	*C*_2_	*C*_3_	*C*_4_	*C*_5_	*C*_6_	*C*_7_	*C*_8_	*C*_9_	*C*_10_	*C*_11_
1	8.8	8.8	0	0	0	0	0	0	0	0
0	0.7	6.8	0	0	0	0	0	0	0	0
1	2.7	6.4	0	0	0	0	0	0	0	0
2	3.4	16.3	0.6	0.6	0	0	0	0	0	0
5	1.5	5.7	0	0	0	0	0	0	0.41	0.41

**Table 2.** Transitions in an IBM for bovine brucellosis at time *t*=*t*_0_+*Δt*.

*C*_1_	*C*_2_	*C*_3_	*C*_4_	*C*_5_	*C*_6_	*C*_7_	*C*_8_	*C*_9_	*C*_10_	*C*_11_
0	0	7.5	0	0	0	0	0	0	0	0
2	0.8	6.8	0	0.9	0	0	0	0	0	0
1	2.8	6.4	0	0	0	0	0	0	0	0
3	3.5	16.3	0	0	0	0.7	0	0	0	0
4	1.6	5.7	0	0	0	0	0	0.8	0	0

### 3.3. Individual-based model algorithm

**Figure 2.**
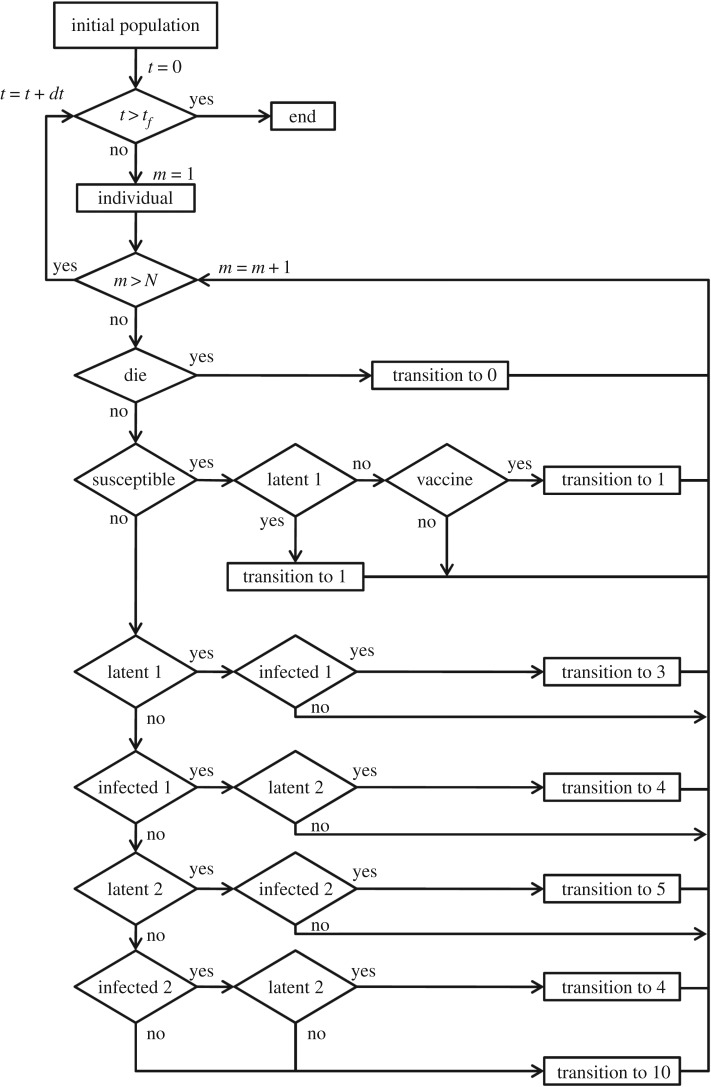
Flowchart of the IBM for bovine brucellosis. The initial population is determined at random. Each individual is evaluated according to formulation described in §(b). The algorithm is ended when the time *t* reaches the maximum value *t*_*f*_. The transitions indicate a change into different category.

Figure 2 shows the flowchart of the IBM for bovine brucellosis. The initial population is determined at random from the given initial conditions. At each instant of time, each subject is considered and found to probability distributions by means of which the transition occurs. After evaluating all *N* individuals, time *t* is increased in *dt*. The algorithm terminates when the final simulation time (*t*_*f*_) reaches a predetermined value. The routines implemented in Scilab 6.0 are available upon request.

**Figure 3.**
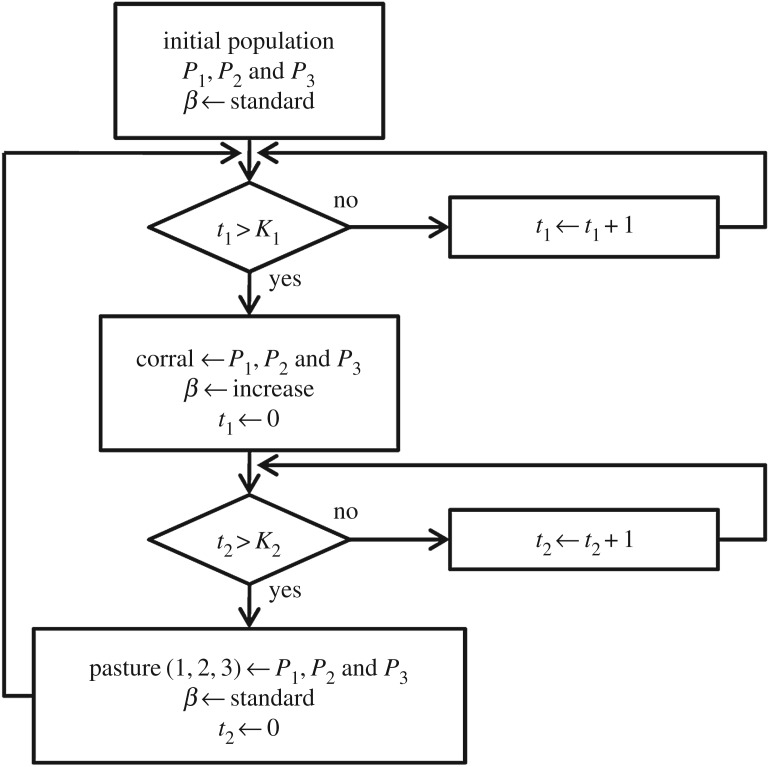
Spatial mobility of individuals. Interaction between animals of different areas. This flowchart shows that after *t*=*K*_1_ all animals were transferred to the corral. After *t*=*K*_2_ the animals return to their pastures. See more details in §(b).

We have also examined the influence of spatial movement of the individuals. Figure 3 represents the movement of individuals in established areas (according to the simulated scenario discussed in the examples).

### 3.4. Control application

Some of the main strategies investigated to control bovine brucellosis are based on continuous vaccination and slaughter of infected animals. Continuous vaccination is understood as an interrupt control action of some specific percentage of animals. Usually, an animal is slaughtered when its infection is confirmed.

However, there are other forms of studying control in bovine brucellosis, such as isolation, pulsed vaccination, optimal control, quarantine and promotion of educational campaigns [28]. In this paper, we have analysed the continuous action with the pulsed vaccination. The pulsed control is usually determined by means of a nonlinear optimization [29]. In this technique, animals receive vaccine at specific intervals of time. We have considered constant the period and proportion of the animals to be vaccinated.

## 4. Simulation examples

### 4.1. Example 1: disease eradication

The purpose of this example is to evaluate the proposed model and analyse it in a scenario compatible with the model [13], which it presents population of *N*=1700, a period *t*_*f*_=20 years, life expectancy of the population *μ*=8 and the infection rate of patients with latent $\gamma =\frac{5}{3}$. The other parameters are: *β*=7.98×10^−2^; $\delta =\frac{1}{6}$ . The initial condition was *S*_0_=0.8*N*, *V*_0_=0, *L*_10_=0.05*N*, *I*_10_=0.05*N*, *L*_20_=0.05*N*, *I*_20_=0.05*N*. At *t*=0, *C*_2_ has been obtained with 0.25*μ*, *C*_4_ with 0.25*γ* and *C*_6_ with 0.25*δ*.

**Figure 4.**
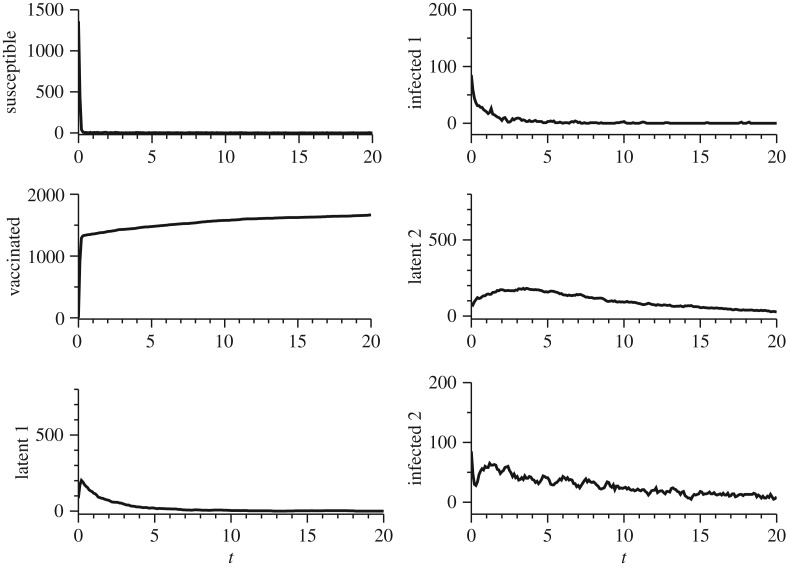
Simulation of IBM for bovine brucellosis. One run of the IBM is shown. It was considered a vaccination rate of *p*=95%. The initial condition was *S*_0_=0.8*N*, *V*_0_=0, *L*_10_=0.05*N*, *I*_10_=0.05*N*, *L*_20_=0.05*N*, *I*_20_=0.05*N*. Other parameters were defined as: *μ*=8, $\gamma =\frac{5}{3}$, *β*=7.98×10^−2^; $\delta =\frac{1}{6}$. At *t*=0, *C*_2_ has been obtained with 0.25*μ*, *C*_4_ with 0.25*γ* and *C*_6_ 0.25*δ*. It is possible to see that with this vaccination rate the number of infected animals (infected 1) approaches to zero. The number of infected female multiparous (infected 2) approaches zero in a longer time due to life expectancy.

**Figure 5.**
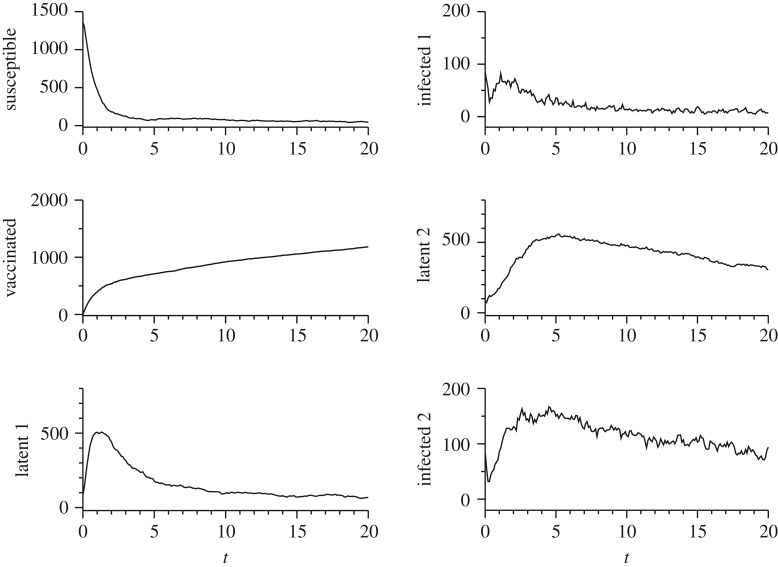
Simulation of IBM for bovine brucellosis. The mean value of 40 runs and standard deviation error are shown. It was considered a vaccination rate of *p*=5%. Initial conditions and other parameters are the same as described in figure 4. This small rate of vaccination is not sufficient to eradicate the disease. The number of infected animals (infected 1) fluctuates around 10.

Figure 4 shows the dynamic behaviour of bovine brucellosis for a vaccination rate of 95%. The applied control has been efficient in eradicating the disease, as one can see the number of infected (infected 1) approaches to zero. The number of infected female multiparous (infected 2) approaches zero in a longer time due to life expectancy. Figure 5 represents the dynamics of the disease at a smaller vaccination rate of 5%. Many seropositive individuals are observed over time. In this case, it is obvious that vaccination at only 5% of the animals has been not sufficient to eradicate the disease.

### 4.2. Example 2: metapopulation effect

In this example, we have considered the spatial distribution of animals. The animals could stay in two different areas: (i) pen or corral, where animals are gathered and collected to a breeding area; (ii) three grazing areas or pasture, where animals are fed with vegetation. With this scenario, we also investigate the effects of dividing the population in small subpopulations. Each group of animals (*P*_1_, *P*_2_ and *P*_3_) is in a pasture and act according to the flowchart presented in figure 2, with infection rate (*β*) standard. After 6 years, all animals are transferred to the corral. In this corral, we have arbitrarily assumed an infection rate 10 times higher than in a pasture due to a small space to move. Animals are not allowed to move around from one pasture to another.

**Figure 6.**
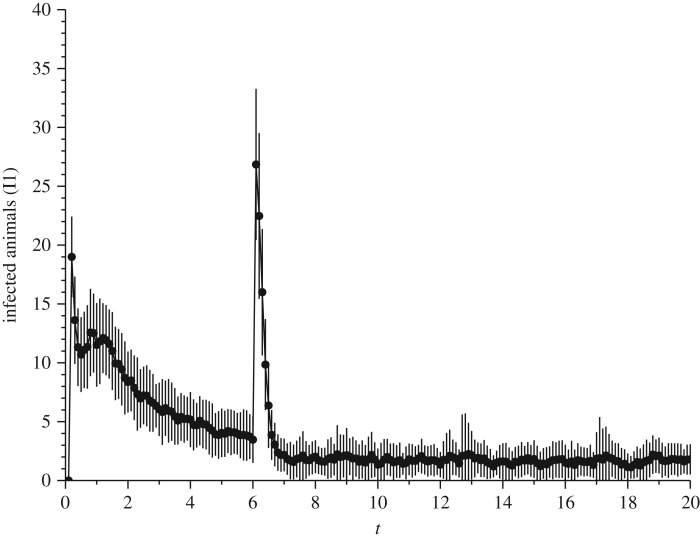
Simulation of spatial IBM for bovine brucellosis. It has been considered a continuous rate of vaccination *p*=75%. The mean value of 40 runs and standard deviation error are shown. The other parameters were: *μ*=8, $\gamma =\frac{5}{3}$ years, *β*=7.98×10^−2^, $\delta =\frac{1}{6}$ years. The initial condition was *S*_0_=0.8*N*, *V*_0_=0, *L*_10_=0.05*N*, *I*_10_=0.05*N*, *L*_20_= 0.05*N*, *I*_20_=0.05*N*, and each pasture started with 400 animals and empty barn. The infection rate in the corral was arbitrarily set to *β*_*barn*_=10*β* at *t*=6 years. This explains the peak of the number of infected.

The model parameters are based on the characteristics of the state of São Paulo [13]. It was considered a population of *N*=1200 for a period *t*_*f*_=20 years. As the life expectancy of the population *μ*=8 years and the infection rate of patients with latent $\gamma =\frac{5}{3}$ years. The other parameters used were: *β*=7.98×10^−2^ and $\delta =\frac{1}{6}$ years. The initial condition was *S*_0_=0.8*N*, *V*_0_=0, *L*_10_=0.05*N*, *I*_10_=0.05*N*, *L*_20_=0.05*N*, *I*_20_=0.05*N*, and each pasture started with 400 animals and empty barn. The areas of pasture and the barn are, respectively, 10 000 and 1200 cells (see §(a), premise 1). The infection rate in the corral was arbitrarily set to *β*_*barn*_=10*β* after 6 years living in the pasture. As was expected, this change of the animal causes a peak in the number of infected, easily seen in figure 6.

### 4.3. Example 3: continuous and pulsed control

**Figure 7.**
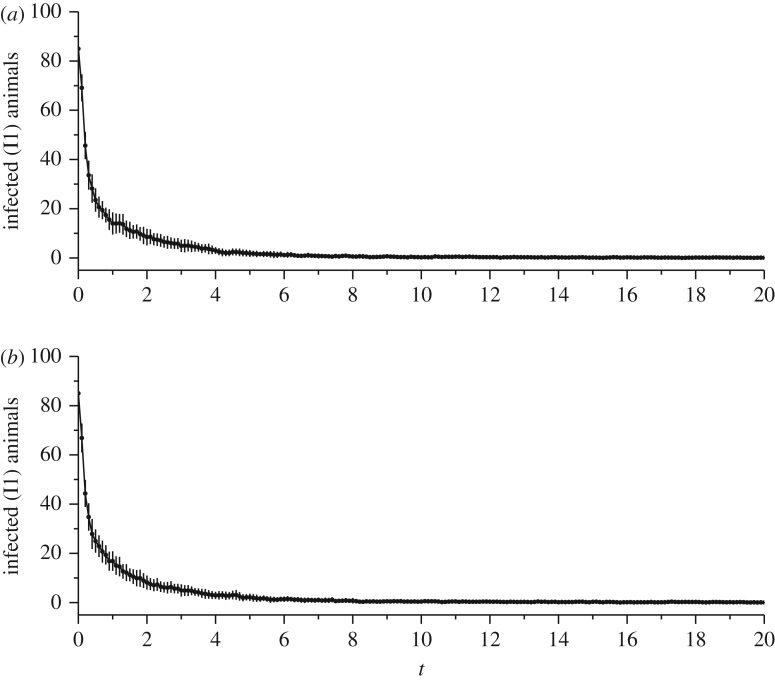
Number of vaccinated females using two different control approaches: continuous (*a*) and pulsed (*b*). The mean value of 40 runs and standard deviation error are shown. The pulses occur in intervals of 0.5 year. Both strategies are efficient to eradicate the diseases, but pulsed control presents a lower financial cost.

This example is an extension of the former. We applied two strategies for population control, continuous monitoring and control pulse (see §(d)). Figure 7 shows the average of vaccinated females for each control method over 40 runs. A standard deviation is also shown. In both cases, it was possible to eradicate the disease. The reduction of standard deviation is also a sign of the robustness of control even in this stochastic simulation environment. However, vaccination pulsed to occur at intervals of 0.5 year, while the continuous vaccination occur in each time step. In the investigated case, it means that the pulsed vaccination is a lower cost, as it spends five time less on vaccines.

## 5. Conclusion

This paper has presented an IBM of bovine brucellosis. We have also presented some control techniques based on continuous and pulsed vaccination. This approach allows the user to deal with a heterogeneous population, spatial distribution and stochastic fluctuations, which are usually neglected in compartmental differential equations framework.

First, we have shown the approach to keep basic dynamics from a compartmental model based on [11,13]. This process has been shown effective to reproduce the mean field behaviour, as seen in [24]. Details of this process, as well as some flowcharts have been given in order to make it easy to reproduce the results presented here. It is important to say that all the routines are written in Scilab, a free software, and they are available upon request.

In the simulation examples, we first presented an illness eradication, in which a rate of 95% of vaccination has been applied to eradicate the disease. This situation may bring some insights to real applications; as has been shown in [29], the level of vaccination may be reduced with a hybrid control, in which the isolation is mixed with the vaccination. This approach can be easily tested using IBM.

The second example has shown the effect of meta-population. This is quite important for the bovine brucellosis case, as the transference of animals among herds is quite common. Again, this strategy may increase our understanding of the difference between big and small number of animals in herds. Nepomuceno *et al.* [24] have shown that a decrease of the size of a herd may significant increase the probability of eradication of a disease. This is another strategy that can be planned in a real situation. Finally, the continuous and pulsed control has been applied to show the successful approach to eradicate a disease. It is important to stress that pulsed vaccination has been effective to eradicate the disease but using a smaller number of vaccinations. This is a typical problem of optimal control that should be addressed in future works.

The IBM is also a suitable framework to test some recent phenomena that have been discussed in the science of network, such as chimera and explosive percolation, which may be used to further investigation of abrupt epidemics [30]. We can also implement different network topologies, such as small-word or random network. This could be particularly interesting to investigate the mean jump length *Δ*, as proposed in [31], as a network alternative parameter to *β*.

## Data accessibility

This article has no additional data.
